# Robotic Submesocolic Left Adrenalectomy: The Evolution of Delbet Approach

**DOI:** 10.1002/rcs.70080

**Published:** 2025-06-17

**Authors:** Monica Ortenzi, Andrea Santini, Andrea Balla, Diletta Corallino, Giovanni Lezoche, Mario Guerrieri, Danila Azzolina

**Affiliations:** ^1^ Clinica di Chirurgia Generale e d’Urgenza Università Politecnica delle Marche Ancona Italy; ^2^ Department of General and Digestive Surgery University Hospital Virgen Macarena Sevilla Spain; ^3^ Hepatobiliary Surgery Division IRCCS San Raffaele Scientific Institute Milan Italy; ^4^ Biostatistics and Clinical Trial Methodology Unit Clinical Research Center DEMeTra Department of Translational Medicine University of Naples Federico II Naples Italy

**Keywords:** adrenalectomy, anterior, laparoscopic, robotic, submesocolic

## Abstract

**Introduction:**

The left adrenal gland is prone to being approached with several access points. This study presents a series of robotic submesocolic left adrenalectomies.

**Materials and Methods:**

Intraoperative and post‐operative outcomes of robotic (RB) and laparoscopic (LP) submesocolic (SM) access to the adrenal gland were compared. Subsequently, these were compared to left adrenalectomy performed using the anterior approach (AT).

**Results:**

Operative time was statistically longer in the LP group (*p* < 0.001). There was no statistical difference for postoperative complications. After the propensity matching, there was a correlation between the BMI and the onset of post‐operative complications (OR = 1.01). The operative time was significantly longer in the AT group both overall (*p* = 0.023) and within the LP procedures (*p* < 0.001), but not in the RB procedures (*p* = 0.386). Length of stay was shorter in the SM group (*p* = 0.024).

**Conclusions:**

The RB SM approach to the left adrenal gland is a safe and feasible.

## 1 Introduction

Since the initial report of laparoscopic adrenalectomy by Gagner et al. in 1992 [1], laparoscopic adrenalectomy has emerged as the gold standard technique for managing adrenal tumours [2], because unlike in other fields, it has rapidly demonstrated its superiority compared to the traditional open approach [3].

This superiority was greatly determined by the intrinsic technical challenges that this type of surgery embodies, derived first of all by the anatomic position of the gland itself [4, 5]. The adrenal gland is not reached in laparotomic surgery without the performance of an extended incision, the positioning of retractor systems and without obtaining a limited and often uncontrolled view of the gland, despite the adoption of all the possible technical countermeasures [6].

Given these drawbacks of the traditional technique, it is not surprising that laparoscopy gained a sudden popularity soon after its first application, and, as a matter of fact, rapidly replaced conventional open procedures as the standard of care for certain diseases [2]. Laparoscopy has undoubtedly shown to be an optimal alternative in respect of the traditional approach to adrenal masses, in terms of safety and post‐operative outcomes [7]. To date, the use of laparoscopic technique is indicated for the treatment of functional adrenal tumours with some limitations determined by the size of the tumour and the suspect for malignancy [7, 8, 9, 10, 11, 12, 13, 14, 15, 16, 17].

However, the approach to the adrenal gland can vary greatly, and it is far from being standardized. The choice of it is mostly left to the surgeon's personal experiences and preferences rather than being supported by robust evidence supporting it [18].

In this scenario, the sub mesocolic approach to the left adrenal gland has been proposed as a viable option if the transabdominal route is the selected one. The first surgeon who proposed this approach was Pierre Delbet in 1912 [19, 20] using open approach. Subsequently, this approach was reported for the first time using the laparoscopic approach by Sardi and Robertson in 1994 and 1995, respectively [21].

Nevertheless, the advancements in the surgical techniques proceeded accordingly to those in the technological field. In 1999, the first robotic adrenalectomy was performed [22, 23, 24, 25]. The robotic system was able to overcome some of the drawbacks of laparoscopy thanks to the articulated instruments’ stereoscopic vision, improved magnification and greater range of motion within a limited working space [23, 24].

Truth has to be said however about the fact that a clear superiority of this technique is yet to be proved and laparoscopic adrenalectomy still remains the gold standard procedure both because of the paucity of comparing literature between the two approaches and of the limited series supporting the robotic technique [25, 26].

We reported the first series of robotic Delbet approaches for left sub‐mesocolic adrenalectomy, hypothesising the possibility of obtaining an enhanced surgical performance using a robotic platform in each type of the possible approaches to the adrenal gland already described for the laparoscopic approach. The primary objective of this study was to investigate the feasibility of the sub mesocolic approach to the left gland using a robotic platform. The secondary endpoint is to compare this approach with the anterior approach performed both laparoscopically and robotically.

## 2 Materials and Methods

Patients were retrospectively enroled from a prospectively maintained database of 640 adrenalectomy. For the purpose of this study, the chosen timeframe was between January 2013, when the first robotic adrenalectomy was performed and August 2024.

The inclusion criteria were adult patients who underwent transabdominal left adrenalectomy, both laparoscopic and robotic. Open procedures were excluded. Patients in which a sub‐mesocolic (SM) approach was adopted were divided into 2 groups (laparoscopic, LP, and robotic, RB) and subsequently compared for intra and postoperative outcomes, focussing in particular on complications rates and length of stay (LOS).

The collected variables were age, gender, body mass index (BMI), indication for surgery, comorbidity, conversion to open surgery, intra and postoperative complications graded according to Clavien Dindo classification, LOS, readmission and reoperation [27].

As a secondary endpoint, the submesocolic approach to the left adrenal gland was compared to the transabdominal anterior route (AT) with mobilisation of the splenic flexure [28].

## 3 Surgical Technique

**FIGURE 1:**
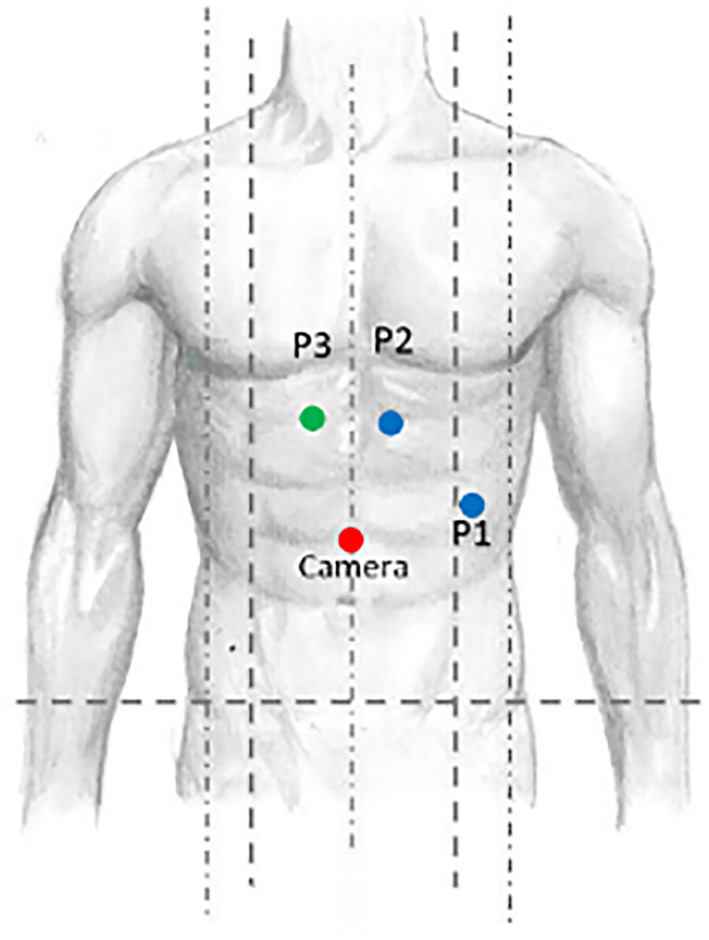
Port position: P1 = vessel sealer; P2 = forceps; P3 = assistant's port.

A robotic skilled general surgeon performed all the operations using the DaVinci Model i Surgical System (Intuitive Surgical Inc. Sunnyvale, CA, USA). The patient was placed supine with abducted legs and in a 45° right side tilt. The optical trocar is inserted supraumbilically on the midline. Two more robotic trocars, one on the left of the midline below the costal arch and one below the XII rib on the anterior axillary line, and one port for the assistant, slightly on the right with respect to the midline and below the costal arch, were inserted Figure 1. The robotic cart was placed between the legs.

**FIGURE 2:**
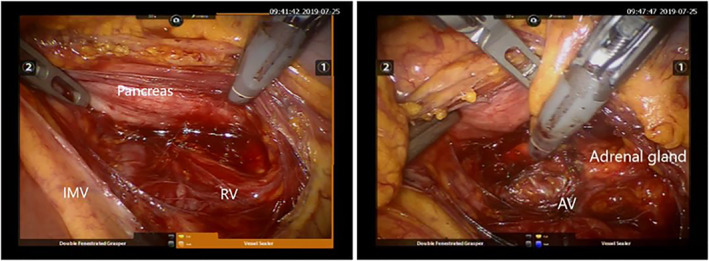
Intraoperative view of the surgical field.

The assistant, through the ancillary port, raised the great momentum and transverse colon. When a good visualisation was obtained, even displacing the first jejunal loop to the right and exposing the inferior mesenteric vein and the superior duodenal recess, the posterior peritoneum was opened by means of a bipolar vessel sealer between the inferior mesenteric vein and duodenal‐jejunal angle to reach and to open the Toldt's fascia. The pancreas was then gently retracted by the assistant and the superior margin of the left renal vein was identified and followed until the origin of the left adrenal vein was identified. The vein was closed with self‐fixating non‐absorbable clips and divided. The gland was then completely mobilised and removed Figure 2. A drain is placed at the end of the operation.

## 4 Statistical Analysis

Statistical analysis has examined associations between surgical approaches and patient outcomes, particularly focussing on complication rates and LOS. The analysis followed several stages to clean and structure data, balance treatment groups, and apply regression models for the outcomes of interest.

Initially, the dataset was cleaned and prepared by excluding patients who underwent an ‘open’ surgical approach to concentrate on minimally invasive ones. Key variables, including weight/BMI, prior interventions, comorbidities, tumour size and re‐interventions, were transformed to binary indicators or parsed as numeric data when appropriate. This preprocessing ensured consistency across variables and standardized the dataset for analysis. Baseline characteristics of patients across different surgical groups were then summarised in a descriptive analysis, which aimed to identify differences in demographic and clinical variables, such as age, BMI, gender, comorbidities and tumour size. Using a group comparison method, we obtained *p*‐values to assess the significance of observed differences across surgical techniques, facilitating an initial overview of potential confounders. Numeric variables were summarised as medians with interquartile ranges, while categorical variables were presented as absolute and relative frequencies. The Wilcoxon test was used for quantitative variables, and the Chi‐Square or Fisher's exact test, as appropriate, was applied for qualitative variables.

To mitigate confounding factors in assessing complications, we applied propensity score weighting. A propensity score model was developed to estimate each patient’s likelihood of undergoing a specific surgical approach based on age, BMI, gender, comorbidities and tumour size. Missing data in predictor variables were managed through multiple imputations, and energy‐balancing weights were applied to improve covariate balance across treatment groups. This approach allowed for a fairer comparison between surgical groups by reducing baseline imbalances. Balance diagnostics have been reported in a plot reporting the mean difference in the covariates before and after the propensity adjustment as a metric [29].

The association between surgical group and complication rates was then examined using Firth’s Bias‐Reduced Logistic Regression model. Though traditional logistic regression is common for modelling binary outcomes, struggles with small datasets and rare events often face issues such as ‘complete separation’ that lead to convergence failures. Firth’s penalised logistic regression offers a robust solution, reducing biases associated with small samples, rare events and separation problems [30].

Finally, to analyse LOS, we employed a generalised linear model (GLM) with a Gamma distribution to handle the skewed nature of LOS data. This model included surgical group and BMI as predictors, allowing for an investigation of how these factors jointly affect LOS. Marginal predictions via AME (Average Marginal Effect) were computed for understanding of how patient characteristics and surgical techniques contribute to variations in hospital stay duration.

Analyses have been conducted with R System 4.3 [31].

## 5 Results

A total of 127 patients were included in this study, of which 72 were in the SM group (58 LP and 14 RB).

In the SM cohort, the two groups (laparoscopic and robotic) statistically differed for age (*p* = 0.016) and BMI (*p* < 0.001) before matching (Table 1).

**TABLE 1:** General characteristics of the patients in the submesocolic approach group.

Variables	SM approach		*p*	Whole cohort (*n* = 72)
	LP (*n* = 58)	RB (*n* = 14)		
Gender [M, *n* (%)]	24 (41.4)	5 (35.7)		29 (40.3%)
Age [years, median IQR]	54.0 [45.0; 63.8]	58.0 [50.2; 64.2]	5.05	55.5 [45.0; 64.0]
BMI [kg/m^2^ median: IQR]	24.6 [22.4; 28.9]	23.5 [22.3; 25.2]	5.10	24.0 [22.4; 28.4]
Comorbidities > 1 [y, *n* (%)]	19 (32.8)	12 (85.7)	NS	31 (43.1)
Previous abdominal surgery > 1 [y, *n* (%)]	23 (41.1)	1 (7.14)	NS	46 (65.7)
Incidentaloma [y, *n*(%)]	28 (50.0%)	6 (42.9%)	NS	58 (47.9)
Size [mean, cm ± SD]	3.00 [1.80; 4.30]	3.95 [2.52; 5.25]	3.38	3.30 [2.00; 4.40]
OT [minutes, median; IQR]	60.0 [50.0; 75.0]	85.0 [76.2; 100]	< 0.001	65.0 [50.0; 80.0]
Additional procedures [*n* (%)]	2 (3.4)	0 (0)	NS	2 (2.8)
Intraoperative complications [*n* (%)]	1 (1.72)	0 (0)	NS	1 (1.72)
Conversion [*n* (%)]	1 (1.72)	0 (0)	NS	4 (3.36)
Post‐operative complications [*n* (%)]	3 (5.2)	2 (14.3)	NS	5 (6.9)
Reintervention [*n* (%)]	0 (0)	0 (0)	NS	0 (0)
LS [days, median; IQR]	3.00 [3.00; 4.00]	3.00 [3.00; 4.00]	0.588	3.00 [3.00; 4.00]
Readmission [*n* (%)]	1 (1.72)	0 (0)	NS	3 (2.36)

Abbreviations: IQR = interquartile range, LOS = length of stay, OT = operative time.

There was a statistically significant difference in the operative time (60.0 [50.0; 75.0] vs. 85.0 [76.2; 100] in the LP and RB groups, respectively, *p* < 0.001). In the LP group, two additional procedures were performed: 1 laparoscopic repair of inguinal hernia and 1 ovariectomy. No intraoperative complications occurred, 1 conversion to open surgery was observed in the RB group. One patient in the LP group had a wound infection.

Hospital stay was not significantly different between the two groups (*p* = 0.824). Three non‐surgical complications occurred in the LP group and 2 in the RB group Table 1.

The robotic approach was applied only in 7 patients in the AT group (*N* = 55), and the whole cohort was compared to the SM approach Table 2.

**TABLE 2:** General characteristics of the patients in the anterior approach group compared with the SM approach.

Variables	Approach		*p*
	AT (*n* = 55)	SM (*n* = 72)	
Gender [M, *n* (%)]	28 (50.9)	29 (40.3)	0.456
Age [years, median; IQR]	59.0 [54.0; 71.5]	55.5 [45.0; 64.0]	0.016
BMI [kg/m^2^, median; IQR]	30.0 [25.6; 33.3]	24.0 [22.4; 28.4]	< 0.001
Comorbidities > 1 [y, *n* (%)]	19 (34.5%)	31 (43.1)	0.001
Previous abdominal surgery > 1 [y, *n* (%)]	37 (67.3)	46 (65.7)	0.067
Incidentaloma [y, *n* (%)]	25 (45.4)	58 (47.9)	0.933
Size [cm, median; IQR]	3.60 [2.70; 5.00]	3.30 [2.00; 4.40]	< 0.001
OT [mean, minutes ± SD]	85.0 [72.5; 105]	65.0 [50.0; 80.0]	< 0.001
Additional procedures [*n* (%)]	2 (5.88)	2 (2.8)	0.501
Intraoperative complications [*n* (%)]	1 (1.8)	1 (1.4)	NS
Robotic [*n* (%)]	7 (12.7)	14 (19.4)	0.317
Conversion [*n* (%)]	3 (6.00)	1 (1.4)	0.174
Post‐operative complications [*n* (%)]	3 (6)	5 (6.9)	0.379
Reintervention [*n* (%)]	2 (3.64)	1 (1.4)	0.682
LS [days, median; IQR]	4.00 [3.00; 6.00]	3 (2.36)	0.024
Readmission [*n* (%)]	2 (3.64)	1 (1.4)	NS

The operative time was significantly longer both overall (*p* = 0.023) and within the AT approach (*p* < 0.001), but not in the RB group (*p* = 0.386). LOS stay was shorter in the SM group (*p* = 0.024).

The significance was maintained in the LP group (*p* < 0.001) Table S1 and not in the RB one (*p* = 0.286) Table S2.

Three post‐operative complications did not occur in the AT group (*p* = 376). Two of these complications required readmission, one due to urinary tract infection and one for haemorrhagic necrotic pancreatitis (*p* = ns).

After the propensity matching (Figure S1), there was a correlation between the BMI and the onset of post‐operative complications (OR = 1.01; 95% CI = 0.90–1.14, *p* = 0.842), while a significantly lower occurrence of complications was observed in the LP SM group (OR = 0.08; 95% CI = 0.01–0.77, *p* = 0.005) Table 3.

**TABLE 3:** Factors correlated to post‐operative complications.

Variables	Complications		*p*
	OR	95% CI	
LP SM	0.08	0.01–0.77	**0.005**
RB SM	0.17	0.01–3.12	0.139
BMI	1.01	0.90–1.14	0.842

Length of stay correlated positively with the patient BMI Figure S2.

## 6 Discussion

Several series and reports already exist describing the application of robotics to adrenal surgery, and the present study aimed to determine the flexibility of the robotic platform to be applied to the multitude of already known laparoscopic approaches to the left adrenal gland.

Traditional laparoscopic surgery is associated with certain limitations, including restricted instrument mobility, two‐dimensional visualisation, and tremor amplification. Consequently, it poses challenges for resecting complex pheochromocytomas. In contrast, the Da Vinci robot offers superior ergonomic characteristics, a high‐definition three‐dimensional field of view, and 7 degrees of freedom in its manipulator arm. These attributes effectively eliminate hand tremors and enable precise anatomical identification, significantly reducing operative complexity.

The results of this study proved the feasibility of the robotic transposition of a well‐known laparoscopic approach to the adrenal gland as the sub‐mesocolic access. To date, there is no consensus on the best route to be preferred for left adrenalectomy.

The sub‐mesocolic approach is less frequently performed, but it can offer several advantages [32, 33, 34, 35]. First of all, it allows the early ligation of the adrenal vein, reducing the risk of catecholamines' spread during organ manipulation. The possibility to explore the abdominal cavity and, if necessary, the possibility of performing a contralateral adrenalectomy without changing the position of the patient are other benefits [34, 35].

In this optic, we demonstrated the possibility to use this approach even when the robotic platform is used and, therefore, the possibility to obtain joined benefits from either the used technique or procedure.

In the literature, a striking difference in perioperative outcomes between laparoscopy and robotic surgery was not demonstrated, even if, as a source of controversy towards the spread of the robotic approach, the latter seemed to imply longer operative times and higher costs [33, 34, 35, 36, 37, 38].

Studies on robotic adrenalectomy reported an intraoperative and postoperative complication rate of 0%–20% and 0%–16%, respectively, with similar rates if the RB technique is compared to the LP one, and even when a higher complication rate was reported in the LP cohort, most of the complications belonged to the Clavien Dindo 1 class, with no differences in terms of severe complications [39].

In this study, we demonstrated a potential benefit of the use of the robotic platform in terms of the occurrence of post‐operative complications and LOS.

The advantages were also maintained in the AT cohort, suggesting that the robotic approach could be beneficial if subsequent to a correct patient selection.

Drawbacks for this approach reside in the increased difficulties in patients with increased abdominal obesity and in the experience and confidence of the operator with this type of access to the adrenal gland.

Nevertheless, it has also been demonstrated that the adoption of robotic surgery is hindered by a steep learning curve and substantial costs [40, 41]. Furthermore, studies have indicated that the absence of tactile feedback during robotic procedures may result in inappropriate tumour traction and inadvertent catecholamine release while increasing the risk of bleeding episodes, cardiovascular instability and intraoperative hypertensive crises [42].

Limitations for this study are represented by the limited robotic cases that were analysed.

## 7 Conclusions

Considering the short operative times and the complications free postoperative course that we observed in our experience, the robotic sub‐mesocolic approach is a safe and valuable option to be considered in the excision of the left adrenal gland. More extended comparative studies are needed to reach more conclusive results.

## Author Contributions

M.O. conceiving, drafting, writing the paper. A.S. data collection. A.B. writing the paper. D.C. final approval. G.L. writing the paper. M.G. final approval. D.A. statistical analysis.

## Consent

Patient consent was obtained at the beginning of the study.

## Conflicts of Interest

The authors declare no conflicts of interest.

## Supporting information

Figure S1

Figure S2

Table S1

Table S2

## Data Availability

Data will be available per request to the corresponding author.
