# Supplementary figures and images for: Robotic Submesocolic Left Adrenalectomy: The Evolution of Delbet Approach
Source: Int J Med Robot. 2025 Jun 17;21(3):e70080. doi: 10.1002/rcs.70080 (PMC12172399; doi:10.1002/rcs.70080)

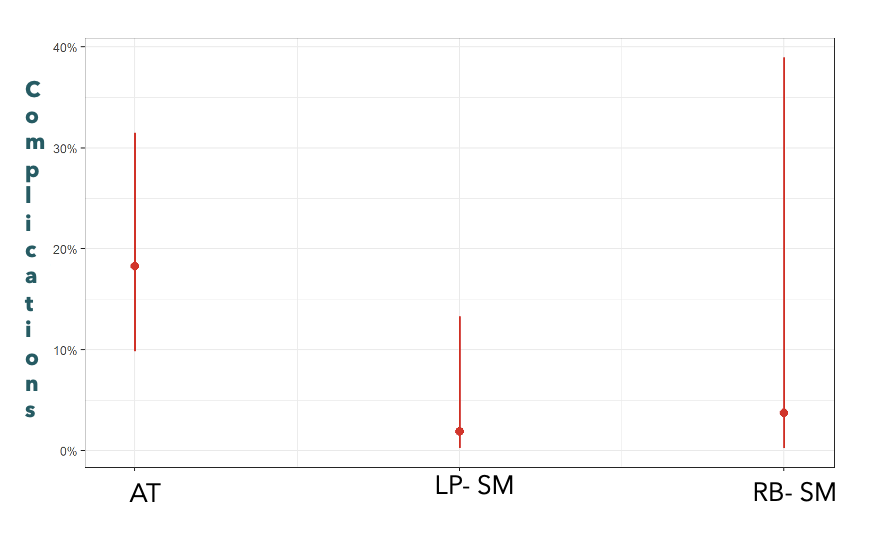


Supplementary Figure 1. Propensity matched analysis for complications

Supplement: Supplementary file 1 — Figure S1 [file RCS-21-e70080-s001.docx]

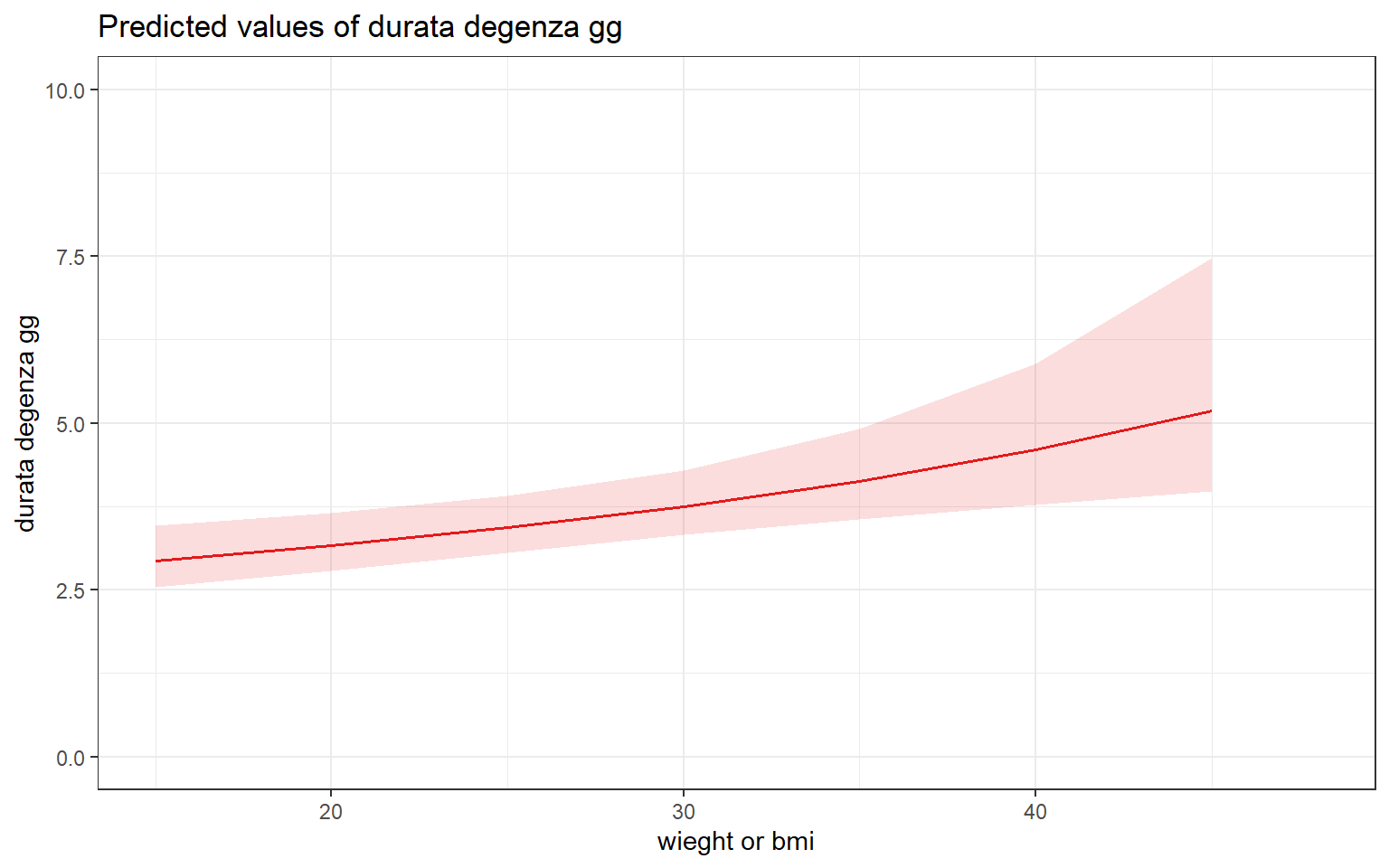


BMI Kg/m2

Supplementary Figure 2. Correlation of the length hospital stay and BMI

Supplement: Supplementary file 2 — Figure S2 [file RCS-21-e70080-s003.docx]
