# Supplementary material for: Robotic Submesocolic Left Adrenalectomy: The Evolution of Delbet Approach
Source: Int J Med Robot. 2025 Jun 17;21(3):e70080. doi: 10.1002/rcs.70080 (PMC12172399; doi:10.1002/rcs.70080)
Supplement: Supplementary file 3 — Table S1 [file RCS-21-e70080-s002.docx]

Supplementary Table 1. General characteristics of the patients in the anterior approach group compared to the SM approach

| **Variables** | **Approach** | | | ***p*** | |
| --- | --- | --- | --- | --- | --- |
|  | **AT LP**  **(n=48)** | **SM LP**  **(n=58)** |  | |  |
| **Gender [M, n(%)]** | 24 (50) | 24 (41.4) | 0.375 | |  |
| **Age [years, median; IQR]** | 61.5 [49.2;73.8] | 54 [39.2;68.8] | 0.002 | |  |
| **BMI [Kg/m^2^, median; IQR]** | 29.7 [22.8;36.5] | 24.6 [18.4;30.79] | 0.010 | |  |
| **Incidentaloma [y, n(%)]** | 22 (45.8) | 25 (43.1) | 0.856 | |  |
| **Size [cm, median; IQR]** | 3.7 [1.9;5.5] | 3 [1.5;4.5] | 0.016 | |  |
| **OT [mean, minutes±SD]** | 85 [61.2;108.8] | 60 [36.2;83.8] | <0.001 | |  |
| **Intraoperative complications [n(%)]** | 4 (8.2) | 0 | 0.025 | |  |
| **Conversion [n(%)]** | 3 (6.2) | 0 | 0.047 | |  |
| **Post-operative complications [n(%)]** | 2 (4.2) | 4 (6.9) | 0.545 | |  |
| **Reintervention [n(%)]** | 0 | 1 (1.7) | 0.368 | |  |
| **LS [days, median;IQR]** | 4 [1,7;6,3] | 3 [09;5.1] | 0.058 | |  |
| **Readmission [n(%)]** | 1 (2) | 1 (1.7) | 0.524 | |  |
