# Supplementary material for: Robotic Submesocolic Left Adrenalectomy: The Evolution of Delbet Approach
Source: Int J Med Robot. 2025 Jun 17;21(3):e70080. doi: 10.1002/rcs.70080 (PMC12172399; doi:10.1002/rcs.70080)
Supplement: Supplementary file 4 — Table S2 [file RCS-21-e70080-s004.docx]

Supplementary Table 2. General characteristics of the patients in the anterior approach group compared to the SM approach

| **Variables** | **Approach** | | | ***p*** | |
| --- | --- | --- | --- | --- | --- |
|  | **AT RB**  **(n=7)** | **SM RB**  **(n=14)** |  | |  |
| **Gender [M, n(%)]** | 4 (57.1) | 5 (35.7) | 0.350 | |  |
| **Age [years, median; IQR]** | 56 [54.2;57.8] | 59 [55.8;60.01] | 0.853 | |  |
| **BMI [Kg/m^2^, median; IQR]** |  |  | 0.003 | |  |
| **Incidentaloma [y, n(%)]** | 4 (57.1) | 8 (57.1) | ns | |  |
| **Size [cm, median; IQR]** | 4.2 [2.53;5.87] | 3.9 [2.16;5,64] | 0.922 | |  |
| **OT [mean, minutes±SD]** | 100 [83.9;116.1] | 85 [58.9;112.1] | 0.286 | |  |
| **Intraoperative complications [n(%)]** | 1 (14.3) | 0 | 0.174 | |  |
| **Conversion [n(%)]** | 0 | 1 (7.1) | 0.469 | |  |
| **Post-operative complications [n(%)]** | 1 (14.3) | 3 (21.4) | 0.694 | |  |
| **Reintervention [n(%)]** | 0 | 0 | ns | |  |
| **LS [days, median;IQR]** | 4.7 [2.9;6.5] | 3.8 [1.7;5.9] | 0.315 | |  |
| **Readmission [n(%)]** | 0 | 0 | ns | |  |
